# Effects of Sublethal Doses of Imidacloprid on Young Adult Honeybee Behaviour

**DOI:** 10.1371/journal.pone.0140814

**Published:** 2015-10-21

**Authors:** Carolina Mengoni Goñalons, Walter Marcelo Farina

**Affiliations:** Laboratorio de Insectos Sociales, Departamento de Biodiversidad y Biología Experimental, IFIBYNE-CONICET, Facultad de Ciencias Exactas y Naturales, Universidad de Buenos Aires, Ciudad Universitaria, Buenos Aires, Argentina; University of North Carolina, Greensboro, UNITED STATES

## Abstract

Imidacloprid (IMI), a neonicotinoid used for its high selective toxicity to insects, is one of the most commonly used pesticides. However, its effect on beneficial insects such as the honeybee *Apis mellifera* L is still controversial. As young adult workers perform in-hive duties that are crucial for colony maintenance and survival, we aimed to assess the effect of sublethal IMI doses on honeybee behaviour during this period. Also, because this insecticide acts as a cholinergic-nicotinic agonist and these pathways take part in insect learning and memory processes; we used IMI to assess their role and the changes they suffer along early adulthood. We focused on appetitive behaviours based on the proboscis extension response. Laboratory reared adults of 2 to 10 days of age were exposed to sublethal IMI doses (0.25 or 0.50ng) administered orally or topically prior to behavioural assessment. Modification of gustatory responsiveness and impairment of learning and memory were found as a result of IMI exposure. These outcomes differed depending on age of evaluation, type of exposure and IMI dose, being the youngest bees more sensitive and the highest oral dose more toxic. Altogether, these results imply that IMI administered at levels found in agroecosystems can reduce sensitivity to reward and impair associative learning in young honeybees. Therefore, once a nectar inflow with IMI traces is distributed within the hive, it could impair in-door duties with negative consequences on colony performance.

## Introduction

Neonicotinoids are widely used as insecticides against pest herbivores [1]. They act as neurotoxins by disrupting the insect nervous system, agonistically activating nicotinic acetylcholine receptors. The high specific affinity to these receptors makes this chemical family of pesticides much more toxic to insects than to mammals [2–4]. Belonging to this group is imidacloprid, which has become one of the most widespread insecticides in the world since the nineties [5, 6]. Its most common method of application in agriculture is as a seed dressing, where the plant takes up the chemical on germination and distributes it systemically as it grows, so that the insecticide is delivered specifically to pests that consume crop tissues [7].

Beneficial insects, such as the honeybee *Apis mellifera* L, can still be harmed as systemic insecticides are potentially present at trace levels in the nectar or pollen of treated plants [7–11]. In fact, IMI has been found in hive food stores [12, 13]. Honeybees are the main pollinators in agricultural systems [14]. IMI is presumably picked up by foragers when gathering resources such as nectar and pollen, which are transported to the hive and stored [11, 15]. The incoming food is rapidly shared within the colony [16, 17] and reaches young adults and larvae.

Honeybees can sense environmental cues when foraging by perceiving different stimuli and establishing associations between them [18]. Individuals that remain inside the hive, such as young workers, can also obtain information from the environment when fed via trophallaxis (mouth to mouth food exchange) with recently collected resources [17, 19] or when feeding on food stored in the hive [20, 21]. These stimuli are mostly of chemosensory modality, such as taste and smell [22, 23]. At early ages of the adult stage, the central nervous system of honeybee workers completes its maturation, where the olfactory system is highly important [24]. Experiences undergone during this period can shape later behaviour. For example, young bees are able to acquire and store rewarded odour information in an associative manner [25, 26], a process that induces long lasting changes in the olfactory circuits of the antennal lobe, which is the first olfactory processing centre in insects. This affects both the processing and the consolidation of odour information [27–30]. Moreover, experiences acquired inside the hive can increase the efficiency of the colony’s foraging related tasks [31, 32]. Therefore, learning induced changes in the brain of young adult bees allow the retrieval of information acquired at very early ages as well as that acquired later in life, in an individual and a social scale [33].

Neonicotinoids act on cholinergic pathways, and acetylcholine plays a major role in insect synaptic transmission. In honeybees, nicotinic acetylcholine receptors are expressed in brain areas associated with mechanosensory antennal information, visual and olfactory processing, learning and memory [34]. IMI impairs associative learning and memory in foraging age honeybees [34–36] and also reduces their gustatory sensitivity [37]. Forager honeybees experience changes in waggle dance communication, orientation and navigation after feeding resources containing IMI traces [37–39]. In the case of young honeybees, IMI has been found to have a dual effect on non-associative learning: contact exposure delays habituation to sucrose in 7-day-old bees, but enhances it in 8-day-old bees [40]. These results suggest changes in the nicotinic pathways throughout the in-hive period of honeybee workers.

As commercial beehives are transported to agricultural settings and remain there for a time to forage pollinator dependent crops, colony individuals of all ages are potentially exposed to agrochemicals. This scenario, together with the fact that during the early adulthood the honeybee is susceptible to stimuli that can shape later behaviour, motivated us to study the effects of IMI on preforaging aged workers. We used doses that were sublethal for forager bees (0.25 and 0.50 ng per bee), where the lowest was also field realistic [11]. We considered both contact and oral exposure. We focused on appetitive behaviours that are based on the proboscis extension response (PER), an innate reflex towards antennal stimulation with sucrose solution [41]. We studied the effect of acute exposure to IMI on gustatory responsiveness [42] and olfactory learning [43], with the purpose of correlating both variables. We considered preforaging bees of different age groups to evaluate if IMI has differential effects depending on the age of exposure. We found IMI effects on both variables, results which differed depending on age of evaluation, type of exposure and IMI dose.

## Materials and Methods

### Study site and animals

The study was carried out during the summer-autumn seasons of 2012 to 2014 in the experimental field of the Facultad de Ciencias Exactas y Naturales of the Universidad de Buenos Aires, Argentina (S 34°32’, W 58°26’). Newly emerged European honeybees (*Apis mellifera* L) were obtained from sealed brood frames taken from the experimental apiary and placed in an incubator at 32°C and 55% RH. After emergence, workers were collected in groups of 60–120 individuals and confined in wooden boxes (10 cm x 10 cm x 10 cm) with a metallic mesh on one side and a plastic door on another. Cages were kept in another incubator at 31°C. They offered 16% w/w sucrose solution and pollen *ad libitum*, and were checked every 1 or 2 days. Food was replaced every 2 or 3 days and dead bees were removed whenever needed. With the purpose of assessing differential effects of IMI traces according to the adult age of exposure, three groups were considered. Therefore, young workers were tested on laboratory bioassays when they were 2/3, 5/6 or 9/10 days old. Experimental bees were anaesthetized at -4°C to minimise suffering and harnessed in carved pipette tips, which restrained body movement, but allowed them to freely move their mouthparts and antennae. Afterwards, they were kept in the incubator until administration of IMI treatments.

### IMI administration

IMI powder was dissolved in dimethyl sulfoxide (DMSO). When IMI was administered topically, harnessed bees were taken out of the incubator after three hours and divided into three experimental groups. A drop of 1 μl of DMSO alone (Control), or with 0.25 or 0.50 ng of IMI was applied on the thorax of each bee. Olfactory PER conditioning started fifteen minutes after IMI administration. When the insecticide was administered orally, harnessed bees were taken out after two hours and fifteen minutes and divided into four experimental groups. Each bee was fed with 3 μl of 16% w/w sucrose solution. One group received sucrose solution alone (DMSO control), another received sucrose solution with DMSO (IMI control) and the remaining experimental groups received the latter plus 0.25 or 0.50 ng of IMI. DMSO concentration in sucrose solution was 0.33% v/v. As dietary exposure meant having to feed the bee with sucrose solution, we therefore chose to administer the treatment one hour before behavioural assays so as to not reduce motivation. Groups receiving the solvent with no IMI served as a control series to assess the effect of the insecticide. In the case of oral administration, and additional series in which bees were fed sucrose solution alone served as a control series to assess the effect of the solvent.

### Olfactory PER conditioning

Bees with both types of IMI administration were submitted to olfactory conditioning. Only bees that extended their proboscis to 50% w/w sucrose solution (unconditioned response towards the unconditioned stimulus) were used for olfactory conditioning. During conditioning, the harnessed bee was placed between a device that produced a constant airflow and an extractor fan which removed released odours. The airstream (2.5 ml s^-1^) was delivered to the head of the bee 2 cm away from it. Bees that responded to the mechanical air stimulus were discarded.

During the training phase, a pure odour was presented paired with 50% w/w sucrose solution (conditioned stimulus). Odour was delivered when, by means of an electric valve, the airflow was redirected to pass through a syringe containing 4 μl of 1-hexanol impregnated on a 30 mm x 3 mm piece of filter paper. Odour was delivered during 6 s and the reward was presented during the last 3 s of this period by touching the antennae with 50% w/w sucrose solution and then feeding the bee. A conditioned response (CR) was computed if the bee fully extended its proboscis during the first 3 s of odour delivery. One trial lasted for 39 s and was composed of 16 s of clean airflow, 6 s of odour and 17 s of clean airflow. Training consisted of 5 trials with an inter-trial interval of 15 minutes. Bees that presented spontaneous PER towards the odour were discarded from analysis. A period of 20 minutes was kept between the last trial and the testing phase, which consisted of a single non-rewarded presentation of the conditioned stimulus. After the testing phase, the unconditioned response was verified and bees that did not present it were discarded from further analyses.

### Sucrose sensitivity

Only bees administered with oral IMI were tested for their sucrose sensitivity. Bees were stimulated with sucrose solutions of increasing concentrations (0.1, 0.3, 1, 3, 10, 30 and 50% w/w) by touching their antennae [42]. The lowest sucrose concentration at which an individual responded by extending its proboscis was interpreted as its sucrose response threshold (SRT). Bees were lined up in groups of 30–40 individuals and tested sequentially for each concentration, i.e. all bees were presented with 0.1% solution first, then with 0.3% solution and so on. Before each sucrose solution presentation, all bees were tested for their response to water (0%). This controlled potential effects of repeated sucrose stimulation that could lead to increased sensitization or habituation, as well as assuring that extension of the proboscis was not due to thirst. The inter-stimulus interval between water and sucrose solution varied between 4–5 minutes depending on the number of individuals tested. At the end of the experiment, a gustatory response score (GRS) was obtained for each bee. This score was based on the number of sucrose concentrations to which the bees responded. The response was arbitrarily quantified with scores from one to seven, where a value of 1 represented a bee that only responded to one concentration of sucrose (50% w/w), while a score of seven represented an individual that responded to all concentrations tested. If a bee failed to respond to one sucrose concentration in the middle of a response series (e.g. responded to 0.3, 3 and 10%, but did not respond to 1%), this 'failed' response was considered to be an error and the bee was deemed to have responded to that concentration as well. A bee that did not respond to any of the sucrose concentrations (score of 0) was excluded from further analyses. In addition, those bees that responded to all sucrose concentrations and all presentations of water were excluded from analyses as they appeared not to be able to discriminate between sucrose solution and water.

### Mortality

Survival of topically exposed bees was assessed 15 minutes and 2 hours after exposure. Bees exposed orally to IMI were fed with 50% w/w sucrose solution and kept in the incubator for 24 hours. Mortality was assessed 2, 20 and 24 hours post-treatment. Bees were considered dead when there were no movements of the antennae or the abdomen.

### Statistical analyses

During conditioning, the outcome variable was the presence of a conditioned response (CR), the extension of the proboscis towards the odour. Comparison of performance during the conditioning between IMI treatments was assessed by means of an ANOVA for repeated measures. Monte Carlo studies have shown that it is permissible to use ANOVA on dichotomous data under certain conditions [44]. The repeated measures were the successive trials and the fixed factor was the IMI group. If we detected statistical differences, we carried out LSD tests to detect differences between groups.

Regarding the testing phase, we compared the proportion of bees that presented a conditioned response between IMI treatments through a homogeneity G test [45]. When significant differences were found, multiple comparisons were performed. For the contact exposure series, we compared all groups. For the oral exposure series, we compared both control groups between each other, both IMI doses between each other and each dose with the IMI control group. When performing multiple comparisons, a Šidák correction was applied to the significance level α (α’ = 1 –(1 – α) ^n^-1^, where *n* stands for the number of comparisons). Effect of IMI treatment on the unconditioned response, the extension of the proboscis towards 50% w/w sucrose solution, was evaluated through Fisher’s homogeneity exact test.

Effect of IMI treatment on sucrose responsiveness, using the Gustatory Response Score, was assessed by means of a one way ANOVA, followed by LSD tests to detect differences between groups. In the case of 2/3-day-old bees, the normality assumption was not fulfilled, so a non-parametric test was applied (Kruskal Wallis ANOVA by ranks).

Comparison of mortality was analysed through an ANOVA for repeated measures in the oral exposure series; while mortality after contact exposure to IMI was compared by means of a Mann-Whitney test. In every case, an analysis was performed for each age group.

In all cases, the significance level used was α = 0.05.

## Results

### Olfactory PER conditioning

**Fig 1:**
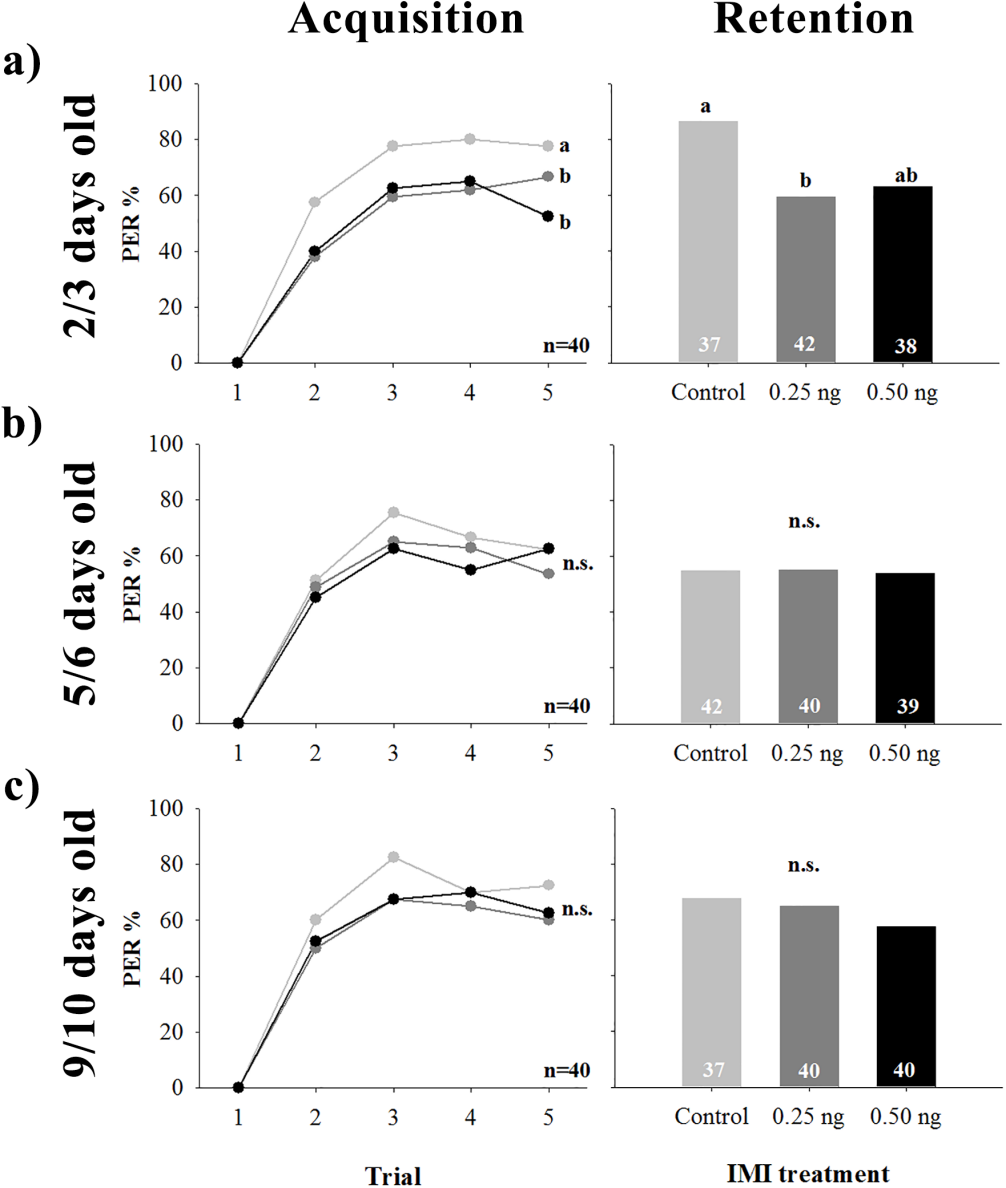
Effect of acute imidacloprid (IMI) contact on olfactory learning and memory of young honeybee workers. Bees were submitted to a classical conditioning protocol fifteen minutes after being topically exposed to 0 (light gray), 0.25 (dark gray) or 0.50 ng (black) of IMI dissolved in DMSO. The percentage of bees that extended their proboscis (PER%) towards the odour was quantified over the course of five training trials (Acquisition, left) and a single testing trial fifteen minutes after training (Retention, right). Individuals used were **a)** 2/3 days old, **b)** 5/6 days old or **c)** 9/10 days old. Numbers inside bars indicate sample size. Different letters stand for statistical differences between IMI treatments (*p* < 0.05).

Bees were submitted to olfactory conditioning fifteen minutes after contact exposure and one hour after oral exposure to IMI. Conditioning consisted of training and testing phases. Contact exposure to IMI had no effect on the percentage of PER during the training phase on 5 day old bees and older, but reduced the performance of 2/3 day old bees (Fig 1, left; two way RM ANOVA, factor IMI; 2/3 days old: F_2,117_ = 3.4259, p = 0.0358; 5/6 days old: F_2,117_ = 0.6831, p = 0.5070; 9/10 days old: F_2,117_ = 1.0369, p = 0.5070). In the latter case, IMI doses were not significantly different from each other. The interaction factor trials x factor IMI was non-significant (2/3 days old: F_6,351_ = 0.5399, p = 0.7777; 5/6 days old: F_6,351_ = 0.4782, p = 0.8245; 9/10 days old: F_6,351_ = 0.2874, p = 0.9428) and there was a significant difference between trials (2/3 days old: F_3,351_ = 10.3592, p<0.0001; 5/6 days old: F_3,351_ = 4.8391, p = 0.0026; 9/10 days old: F_3,351_ = 5.1831, p = 0.0016). When testing for memory retention fifteen minutes after training, contact IMI exposure reduced the percentage of PER in 2/3 day old bees (Fig 1A, right; Homogeneity G test, G = 7.611, p = 0.0222). There was no effect on memory retention of older bees (Fig 1B and 1C, right; Homogeneity G test; 5/6 days old: G = 0.0118, p = 0.9941; 9/10 days old: G = 1.2074, p = 0.5468).

**Fig 2:**
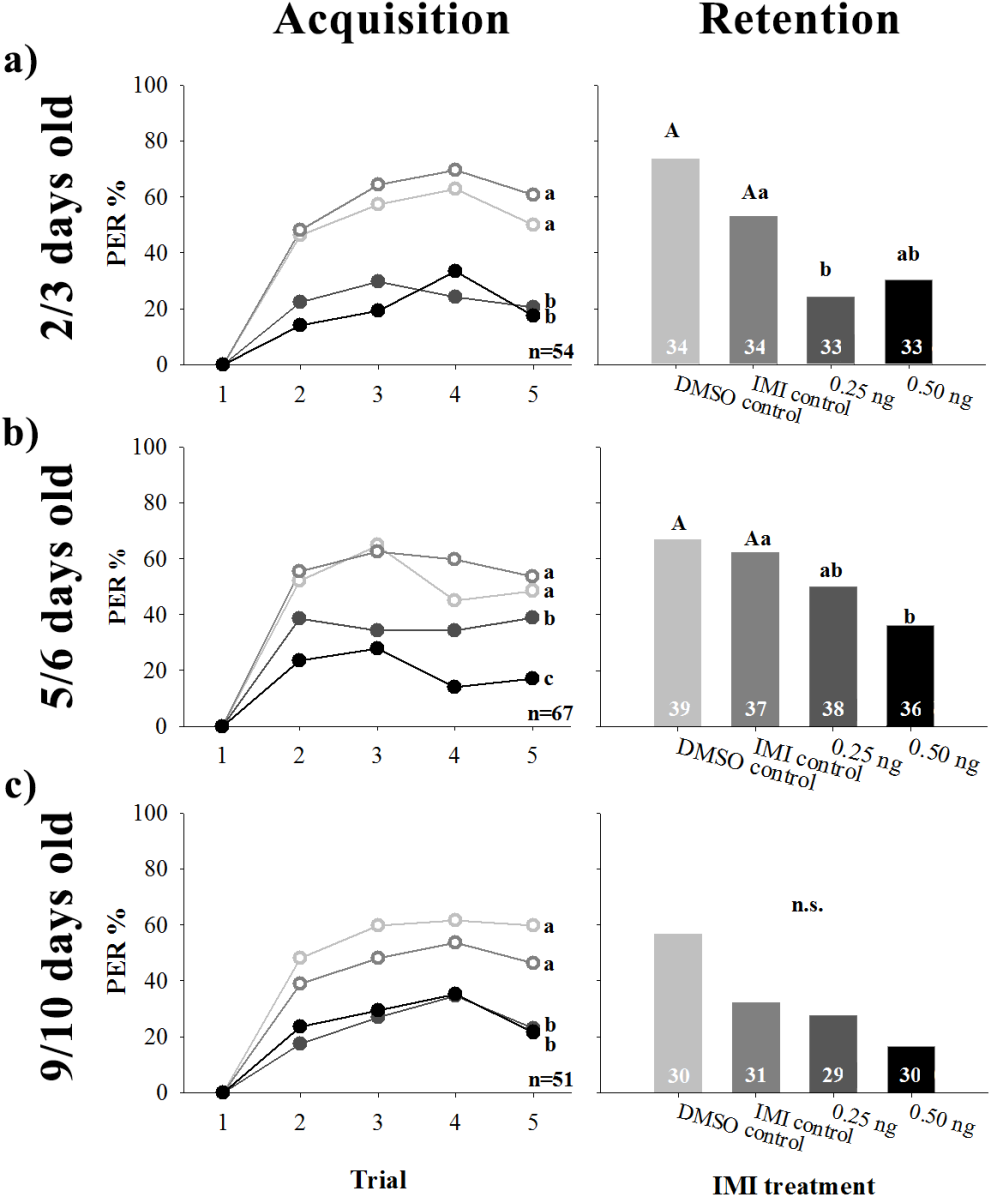
Effect of acute imidacloprid (IMI) ingestion on olfactory learning and memory of young honeybee workers. Bees were submitted to a classical conditioning protocol one hour after being fed with sucrose solution alone (DMSO control; light gray, hollow circles) or with 0 (IMI control; dark gray, hollow circles), 0.25 (darker gray, filled circles) or 0.50 ng (black, filled circles) of IMI dissolved in DMSO. The percentage of bees that extended their proboscis (PER%) towards the odour was quantified over the course of five training trials (Acquisition, left) and a single testing trial fifteen minutes after training (Retention, right). Individuals used were **a)** 2/3 days old, **b)** 5/6 days old or **c)** 9/10 days old. Numbers inside bars indicate sample size. Different letters stand for statistical differences between IMI treatments (*p* < 0.05).

Oral exposure to IMI reduced the percentage of PER in all trials of the training phase on bees of all ages (Fig 2, left; two way RM ANOVA, factor IMI; 2/3 days old: F_3,212_ = 22.1425, p<0.0001; 5/6 days old: F_3,262_ = 20.0347, p<0.0001; 9/10 days old: F_3,183_ = 13.7136, p<0.0001). The interaction factor trials x factor IMI was non-significant (2/3 days old: F_9,636_ = 0.9503, p = 0.4805; 5/6 days old: F_9,786_ = 1.1017, p = 0.3589; 9/10 days old: F_9,549_ = 0.2405, p = 0.9884) and there was a significant difference between trials (2/3 days old: F_3,636_ = 6.4657, p = 0.0002; 5/6 days old: F_3,786_ = 3.2533, p = 0.0212; 9/10 days old: F_3,549_ = 4.4828, p = 0.0040). In the case of 2/3 and 9/10 day old bees, application of both IMI doses resulted in the same level of acquisition impairment. Instead, bees of intermediate ages seemed to be affected by IMI in a dose-dependent manner (Fig 2B, left). Oral IMI exposure reduced the percentage of conditioned response in memory retention tests of bees up to 6 days of age, while it had no effect on older bees (Fig 2, right; Homogeneity G test; 2/3 days old: G = 21.333, p = 0.0001; 5/6 days old: G = 9.244, p = 0.0262; 9/10 days old: G = 11.528, p = 0.0092). In the latter case, the significant effect observed in the overall homogeneity G test was not substantiated when performing post-hoc comparisons (all of them resulted non-significant). In younger bees, ingestion of 0.25 ng prior to olfactory conditioning was enough to impair memory retention. The higher dose, though resulting in similar PER values as the lower dose (24.2 and 30.3%, respectively), was statistically not different from the IMI control group. On the contrary, in 5/6 day old bees, the higher dose was the one that reduced CR. This result is consistent with the effect observed in the acquisition phase. In bees of all ages, ingestion of the solvent DMSO had no effect on associative learning.

### Sucrose sensitivity

**Fig 3:**
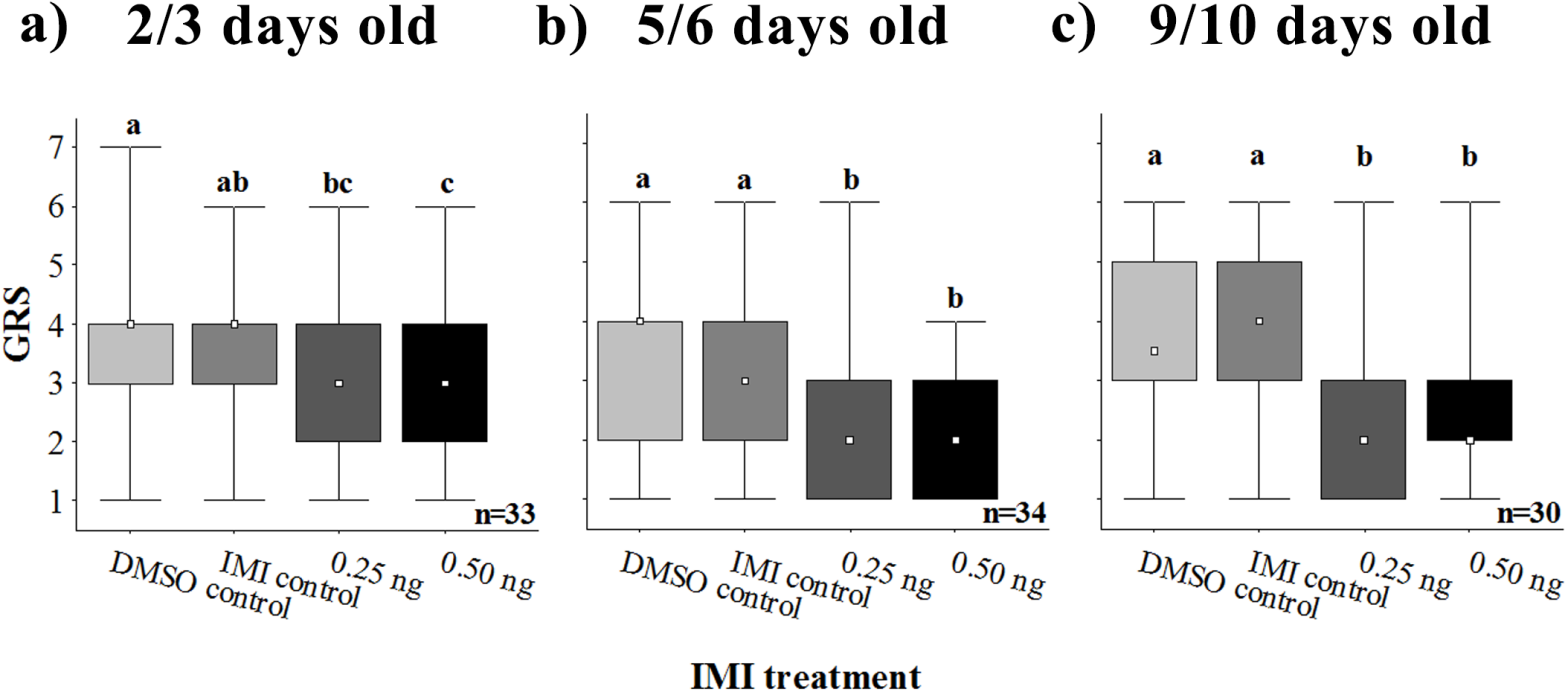
Effect of acute imidacloprid (IMI) ingestion on sucrose sensitivity of young honeybee workers. Bees were tested for their sensitivity to sucrose one hour after being fed with sucrose solution alone (DMSO control, light gray) or 0 (IMI control, dark gray), 0.25 (darker gray) or 0.50 ng (black) of IMI dissolved in DMSO. Gustatory Response Scores (GRS) were calculated for each bee of **a)** 2/3, **b)** 5/6 and **c)** 9/10 days of age. The square, box and whiskers represent the median, inter quartile interval and data range, respectively. Different letters stand for statistical differences between IMI treatments (*p* < 0.05).

Bees were tested for their sucrose response threshold one hour after oral exposure to IMI. Ingestion of the solvent DMSO did not affect sensitibity to sucrose in any age group (Fig 3, light vs. dark gray bars). On the contrary, ingestion of the insecticide affected gustatory responsiveness of bees of all ages, raising the sucrose response threshold (2/3 days olds: Kruskal Wallis test, H_3,132_ = 17.0183, p = 0.0007; 5/6 days olds: one way ANOVA, F_3,132_ = 7.7042, p<0.0001; 9/10 days old: one way ANOVA, F_3,116_ = 8.4136, p<0.0001). Bees of 5 days of age and older receiving 0.25 or 0.50 ng of IMI (darker gray and black bars, respectively) presented lower gustatory response scores (GRS) compared to control bees (IMI control, dark gray bars), with no significant differences between IMI doses. In the case of younger bees, both doses decreased GRS, but only the highest one was significantly different from the control group.

### Mortality and sensory-motor skills

Survival of bees used during the PER procedures were measured when they were 2/3, 5/6 or 9/10 days old. To assess if the doses used were in fact sublethal in this age interval, we kept bees harnessed after IMI administration and evaluated their survival. Bee mortality was measured 15 minutes and 2 hours after contact exposure and 2, 20 and 24 hours after oral exposure. IMI applied topically did not affect the number of live bees in any of the age groups (Table 1). Considering all ages, survivorship of control bees reached up to 83.05%, and that of treated bees ranged between 83.7 and 87.3%. When bees ingested IMI, their survival did not differ from control bees even 24 hours after administration, in every age group assessed (Table 1). At this moment, survivorship of control bees reached up to 57.1 and 60.0% (DMSO and IMI controls, respectively) and that of treated bees ranged between 51.4 and 80.6%.

**Table 1:** Survival of young honeybee workers after acute exposure to imidacloprid (IMI).

				Survival (%)					
Exposure	Age group	IMI treatment	n	15 minutes	2 hours	20 hours	24 hours	F	*p* value
Contact	2/3	Control	79	93.67	86.08	-	-	F_2,234_ = 0.013	0.9868
		0.25 ng	79	92.41	87.34	-	-		
		0.50 ng	79	91.14	87.34	-	-		
	5/6	Control	71	97.18	87.32	-	-	F_2,207_ = 0.387	0.6799
		0.25 ng	75	97.33	86.67	-	-		
		0.50 ng	70	90.00	87.14	-	-		
	9/10	Control	59	93.22	83.05	-	-	F_2,162_ = 0.020	0.9800
		0.25 ng	55	94.55	83.64	-	-		
		0.50 ng	57	94.74	84.21	-	-		
Oral	2/3	DMSO control	39	-	100.00	76.92	66.67	F_3,140_ = 0.572	0.6340
		IMI control	36	-	100.00	77.78	66.67		
		0.25 ng	36	-	100.00	86.11	80.56		
		0.50 ng	36	-	100.00	77.78	69.44		
	5/6	DMSO control	59	-	100.00	81.36	77.97	F_3,232_ = 0.646	0.5860
		IMI control	67	-	100.00	83.58	82.09		
		0.25 ng	72	-	98.61	84.72	77.78		
		0.50 ng	69	-	98.55	79.71	75.36		
	9/10	DMSO control	35	-	97.14	65.71	57.14	F_3,128_ = 0.278	0.8410
		IMI control	35	-	97.14	68.57	60.00		
		0.25 ng	35	-	100.00	74.29	51.43		
		0.50 ng	33	-	100.00	72.73	66.67		

Harnessed bees were exposed to IMI either by contact or orally. Individuals used were 2/3, 5/6 or 9/10 days of age. When bees were topically exposed to IMI, survival was measured 15 minutes and 2 hours after exposure. When they were orally exposed to IMI, survival was measured 2, 20 and 24 hours after exposure. The *p*-values indicate significance of the factor IMI (two way RM ANOVA).

To verify that these IMI doses did not affect sensory or motor skills involving the extension of the proboscis in response to the presentation of 50% w/w sucrose solution to the antennae, we assessed this unconditioned response after the conditioning procedure. Neither contact exposure nor ingestion of IMI affected the percentage of PER (Fisher’s exact test; contact exposure, 2/3 days old: p = 0.0.1290, 5/6 days old: p = 0.4740, 9/10 days old: p = 0.8050; oral exposure, 2/3 days old: p = 0.8620, 5/6 days old: 0.7820, 9/10 days old: 0.2770). Without distinguishing age, values of unconditioned response were 85.9, 97.7 and 97.0% in the contact exposure series (control, 0.25 ng and 0.50 ng, respectively), and 96.1, 96.8, 98.0 and 92.95% in the oral exposure series (DMSO control, IMI control, 0.25 ng and 0.50 ng, respectively).

## Discussion

We set out to study the effect of exposing young honeybee workers to imidacloprid, the most common neonicotinoid insecticide used worldwide, on appetitive behaviours. These bees are colony members that show high behavioural and physiological plasticity [33] and perform relevant tasks within the hive that guarantee nest maintenance and care [46, 47]. According to the evidence gathered in this study, we can conclude that the chosen doses of IMI indeed affect gustatory responsiveness and olfactory learning in young honeybees. The impaired behavioural responses found after exposure to sublethal IMI doses are age dependent, which suggests that the cholinergic nicotinic pathways suffer strong modifications during early honeybee adulthood. This hypothesis was proposed for the first time when studying effects of IMI and its metabolites on habituation of young honeybees [40, 48]. Recently emerged bees (2/3 days of age) were the most affected by any IMI treatment. Bees of the all age categories demonstrated similar learning performances during PER conditioning. Nevertheless, 5/6 day old subjects showed these could be achieved with higher sucrose response thresholds (lower GRS values).

When applied topically, IMI had an effect only on conditioning of the youngest bees, in both training and testing phases. Oral exposure to IMI reduced the percentage of PER, but its effect was more pronounced than contact exposure. While 0.5 ng of contact IMI reduced PER% in the fifth trial from 77.50% to 52.50% compared to the control group, the same dose administered orally produced a higher deficit (from 60.71% to 17.54%, respectively). Dermal application of IMI had no effect on 5 day old bees or older, but oral administration reduced PER values in all age groups. This differential effect is expectable as oral toxicity is greater than dermal toxicity [49, 50], and may be related to the rapid movement of IMI into the digestive system, whereas contact application relies on passive absorption. What is more, oral administration represents a more realistic situation for honeybees as they forage in agricultural ecosystems exposed to the pesticide, ingesting the resources and sharing them with the colony.

When comparing different age classes, it would seem that 2/3 day old bees are more vulnerable to IMI as they are the only age group to be affected by the dermal treatment. In addition, the lower oral dose was sufficient to significantly reduce memory retention in bees of this age class, while 5/6 day old bees were affected by the highest dose and 9/10 day old bees showed no alterations in CR due to IMI exposure. This last observation would lead to inferring that sensitivity to IMI declines with age. In accordance with this hypothesis, both doses of oral IMI equally reduced response in the training phase of 2/3 day old bees, while 5/6 day old bees were affected by oral IMI in a dose dependant manner. Nevertheless, the eldest age group was affected by IMI during the training phase in a similar way as the youngest group. Although we cannot discard a possible effect of the solvent on the performance of 9/10-day-old bees, it is worth remarking that previous reports working with laboratory reared worker bees within the 5 to 8 day old age category showed better retention to an odour rewarded stimulation than those bees of 9–12 days of age [25].

**Fig 4:**
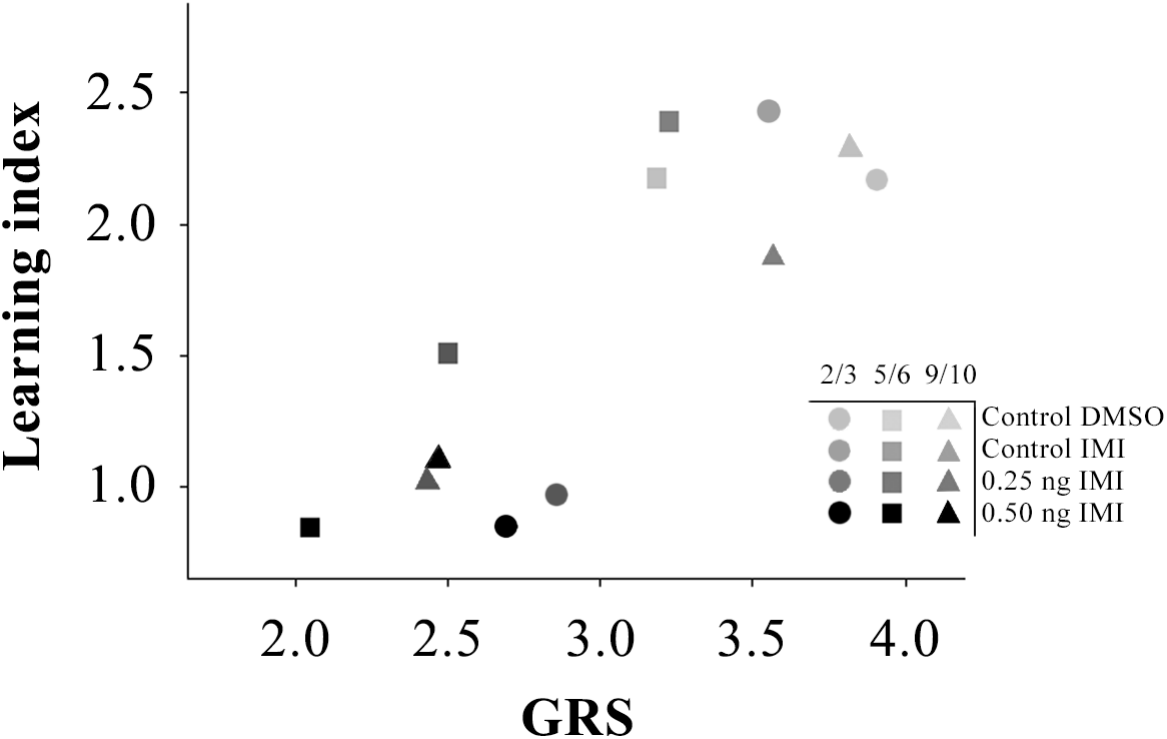
Relationship between learning performance and sucrose sensitivity of young honeybee workers after having ingested IMI. Gustatory Response Scores (GRS) plotted against Learning Indexes (LI) obtained during the acquisition phase of the olfactory PER conditioning. Values of each variable correspond to the mean of different groups of bees. Each symbol represents the combination of GRS and LI values for two groups under the same experimental conditions Age group x IMI treatment.

IMI treatment did not affect the percentage of the unconditioned response to 50% w/w sucrose solution either before, during or after conditioning of bees of any age. Nevertheless, IMI ingestion did affect gustatory responsiveness by raising the sucrose response threshold. This means that 50% w/w sucrose solution does not have the same relative value for bees receiving different treatments. For a bee with a high response threshold, a highly concentrated sugar solution is undervalued in contrast with a bee with a lower one. In this sense, sensitivity to reward depends on the bee`s response threshold. One would expect that a high reward promotes a strong association with the conditioned stimulus, and *vice versa* [51]. Therefore, bees with a low SRT would perform better in an olfactory conditioning procedure than bees with a high SRT. This is what be obtained when weighing sucrose responsiveness against learning performance (Fig 4). A learning index (LI) was calculated for each bee as the sum of CR throughout the conditioning phase. As each bee was assessed for only one variable, a correlation analysis could not be performed. However, taking all ages into consideration, results show that control bees presented high GRS and LI while IMI treated bees presented low GRS and low LI. In other words, low sucrose responsiveness due to IMI administration was associated with a deficit in learning performance. This type of correlation has been found in other studies, where bees that presented low SRT showed better memory retention [52, 53]. Therefore, deficits in both acquisition and retention of olfactory memories are accompanied, if not explained by, changes in sensitivity to reward.

Apart from this, it is worth noticing that when comparing between age categories within the same IMI group (different shapes, same gray scale), the lowest GRS values usually belong to the 5/6-day-old bees (squares) even though showing similar LI values (Fig 4). In other words, bees from this intermediate age category achieve similar learning performances despite having higher sucrose response thresholds. This issue suggests that chemosensory responsiveness and learning abilities for hive aged bees are age dependent, but this relation is not linear. In this sense, the ongoing development of the olfactory nervous system during the first days after adult emergence [24, 54, 55] could explain, at least in part, the non-linear changes found here and in other related studies [33].

Once ingested, IMI is metabolised and, together with its metabolites, is differentially distributed throughout the body of the honeybee [49]. Also, these metabolites differ in toxicity [49]. In this study it is not possible to determine if the effects observed are due to imidacloprid or to one of its metabolites as the delays between administration and evaluation differ according to the type of exposure and as assays have a prolonged duration. Nevertheless, the aim of this study was not to assess the toxicity and kinetics of the one compound imidacloprid, but rather investigate the effect of this neonicotinoid present in agroecosystems during a long period.

Ingestion of IMI with nectar is a plausible way of incorporating the insecticide in an exposed agricultural environment. Honeybee foragers exposed to 0.15 ng of IMI—a dose of IMI equivalent to the one used in this study—continue foraging normally, whereas 10 times more IMI is necessary to reduce foraging activity and prolong foraging flights during the first three hours after treatment [15]. In other words, the IMI doses used in this work would not only be unperceived by foragers, but would also not alter their foraging activity. Consequently, entry of contaminated nectar would persist. Within the hive, the gathered material is stored and processed to honey, bee bread, royal jelly and wax [20]. Hence, IMI would accumulate inside the hive. Therefore, even though only the lowest dose chosen in this study is field realistic in terms of a forager’s nectar load [11], in-hive doses could be much higher. The food resources are consumed by worker bees, processed and fed by nurse bees to other workers, the queen, the larvae and the drones. Therefore, contaminated nectar reaches a considerable number of individuals as it is quickly distributed within the colony [17]. Young workers, whose tasks are restricted to the hive, would be exposed exclusively to the contaminated nectar circulating among hive mates or that which is stored in combs. Since the chosen IMI doses could represent in-hive doses and both types of IMI exposure used did not affect mortality in any of the age groups, they constitute a plausible scenario of the agricultural setting’s surroundings.

Many studies have evaluated the effect of this pesticide on honeybees focussing on behaviour of foraging adult workers [35, 37–39, 56, 57]. However, only a few studies [40, 58] besides this one have examined how this neonicotinoid affects behaviours in young adult workers, who are commonly involved in tasks that require coordination among hive mates. In this sense, a deficit in learning performance would have an effect on chemosensory information propagation as well as on nectar distribution inside the hive. Higher sugar response thresholds suggest there could be less nectar distribution [59]. Also, once nectar is distributed there could be difficulties in establishing odour rewarded associations, which leads to inefficient food related information propagation [53]. This would affect not only the distribution of the contaminated nectar itself, but also that of other nectars that are gathered simultaneously. Eventually, foragers overall would be motivated to leave the hive in need to exploit new food sources [60, 61] as gustatory responsiveness of preforager bees are associated with foraging choices later in life [59]. This would imply that hives could face the end of the season with limited and potentially contaminated food reserves. Therefore, this study suggests that more attention should be paid to the effects of commonly used pesticides on young honeybee behaviour, an issue that might explain, at least in part, the observed colony disorganisation reported in honeybee population during the last decades.
